# Asc-1 Transporter (SLC7A10): Homology Models And Molecular Dynamics Insights Into The First Steps Of The Transport Mechanism

**DOI:** 10.1038/s41598-020-60617-y

**Published:** 2020-02-28

**Authors:** Afaf Mikou, Alexandre Cabayé, Anne Goupil, Hugues-Olivier Bertrand, Jean-Pierre Mothet, Francine C. Acher

**Affiliations:** 1 Laboratoire de Chimie et Biochimie Pharmacologiques et Toxicologiques, UMR 8601 CNRS, Université de Paris, 45 rue des Saints-Pères, 75270 Paris Cedex 06, France; 2 BIOVIA, Dassault Systèmes, 10 rue Marcel Dassault, CS 40501, 78946 Vélizy-Villacoublay Cedex, France; 3 LuMIn FRE2036, Université Paris-Saclay, CNRS, ENS Paris-Saclay, CentraleSupélec, 91190 Gif-sur-Yvette, France

**Keywords:** Computational models, Diseases of the nervous system, Transporters in the nervous system

## Abstract

The alanine-serine-cysteine transporter Asc-1 regulates the synaptic availability of d-serine and glycine (the two co-agonists of the NMDA receptor) and is regarded as an important drug target. To shuttle the substrate from the extracellular space to the cytoplasm, this transporter undergoes multiple distinct conformational states. In this work, homology modeling, substrate docking and molecular dynamics simulations were carried out to learn more about the transition between the “outward-open” and “outward-open occluded” states. We identified a transition state involving the highly-conserved unwound TM6 region in which the Phe243 flips close to the d-serine substrate without major movements of TM6. This feature and those of other key residues are proposed to control the binding site and substrate translocation. Competitive inhibitors ACPP, LuAE00527 and SMLC were docked and their binding modes at the substrate binding site corroborated the key role played by Phe243 of TM6. For ACPP and LuAE00527, strong hydrophobic interactions with this residue hinder its mobility and prevent the uptake and the efflux of substrates. As for SMLC, the weaker interactions maintain the flexibility of Phe243 and the efflux process. Overall, we propose a molecular basis for the inhibition of substrate translocation of the Asc-1 transporter that should be valuable for rational drug design.

## Introduction

**Figure 1:**
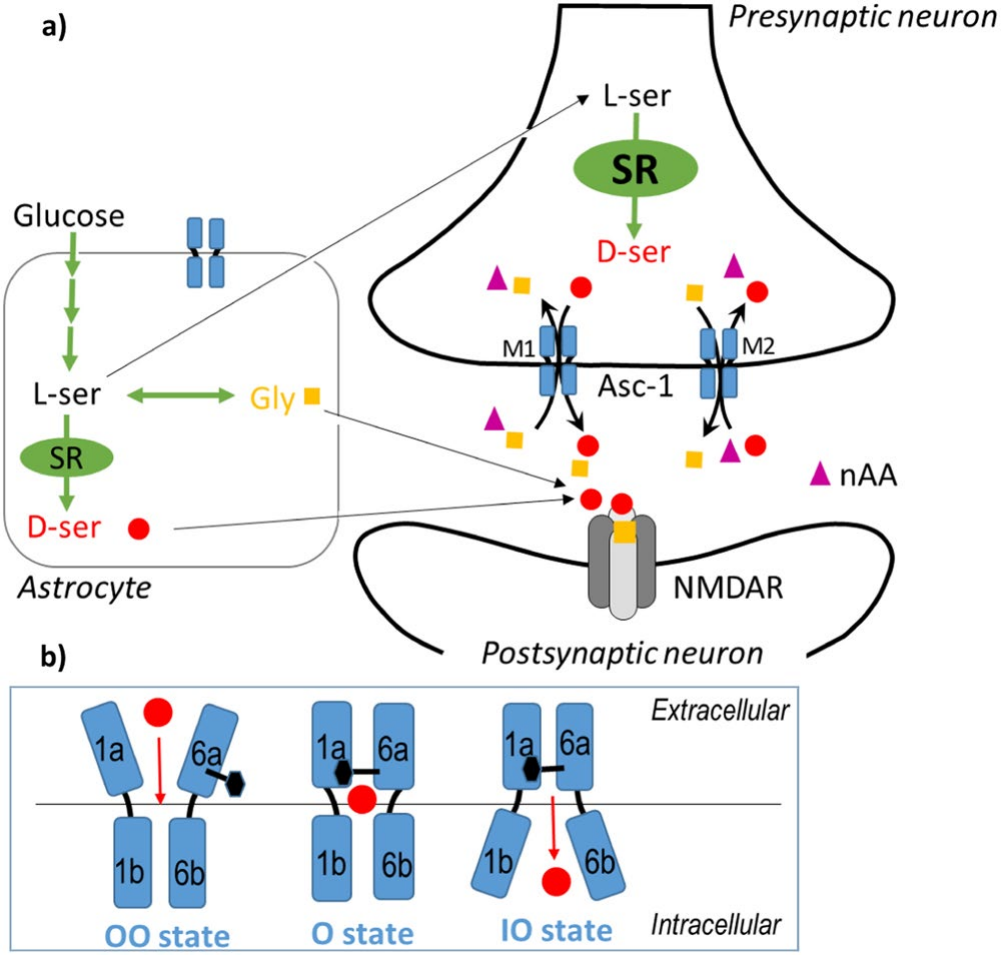
Central role of Asc-1 at the tripartite glutamatergic synapse. (**a**) It has been reported that the Asc-1 transporter (blue) plays an important role in regulating NMDAR-dependent synaptic activity by mediating release of d-serine (red) in exchange to other neutral amino acids (violet) including glycine (yellow). Activation of the GluN1-GluN2 containing NMDA receptors (NMDAR, grey) in addition to glutamate requires the binding of a co-agonist d-serine and/or glycine. Interestingly, the binding of glycine overlaps with the d-serine binding at synaptic NMDARs. Also, d-serine is formed from l-serine by Serine Racemase (SR, green). Transport of d-serine including the efflux of the substrate (indicated by M1 mechanism) along with the uptake of the substrate (indicated by M2 mechanism) across the membrane involves roughly three states: outward-open (OO), occluded (O) and inward-open (IO) (Fig. 1b) with additional intermediate states such as outward-open occluded (OOO) and inward-open occluded (IOO) (not shown).

The alanine-serine-cysteine transporter (referred to as SLC7A10 or Asc-1) is the light chain of the heterodimer amino acid transporter (HAT), and is a Na^+^-independent antiporter distributed throughout the central nervous system (CNS). Asc-1 is present at both astrocytes and neurons^1–3^, and displays high affinity for neutral amino acids and uses diverse substrates, such as l-serine, l-alanine, l-cysteine along with glycine and d-serine^4^. However, glycine and d-serine serve as two necessary and positive allosteric modulators of the N-methyl-d-aspartate (NMDA) subtype of glutamate receptors, and bind to the strychnine insensitive binding site (referred to as the ‘glycine site’) of those receptors (Fig. 1a)^5,6^. For these reasons, Asc-1 is a promising druggable target relevant for treating cognitive affections during normal or pathological aging but also in disorders like schizophrenia^7–12^. Indeed, Asc-1 primarily mediates the efflux of d-serine and glycine through the hetero-exchange with other neutral amino acids, and thus regulates glutamatergic neurotransmission and synaptic plasticity in the forebrain^13^. But, recent studies suggest that Asc-1 is also involved in the control of glycinergic transmission in the caudal brain and spinal cord^2,3,14^. SLC7a10-null mice display reduced levels of glycine, but not d-serine, and show rigidity and myoclonus characteristic of the clinical condition called hyperekplexia^2,14^.

Given the important physiological function of Asc-1 in regulating d-serine and glycine levels, modulating Asc-1 function could provide therapeutical benefits for alleviating the symptoms caused notably by the hypofunction of NMDARs in schizophrenia^15–17^. However, the preferred direction of the d-serine and glycine transport mode by Asc-1 *in vivo* remains uncertain to date. Although some inhibitors decrease the tonic release of d-serine and impair NMDAR functions, others prevent d-serine uptake in cells^8,16,18,19^. SLC7a10-null mice show reduced glycinergic inhibition and NMDAR-mediated glutamatergic transmission but increased GABAergic neurotransmission caused by the reduced levels of glycine^2,14^.

Therefore, elucidating the transport mechanism of Asc-1 is central for our basic understanding of the function of this critical transmembrane protein. However, many details of the mechanisms implicated in the regulation of Asc-1 and its shuttle of amino acids across cell membranes remain unclear.

Unfortunately, atomic-level intricacies of human Asc-1 regulation and shuttle properties remain elusive as no X-ray crystallographic structure has been published to date. Although, the bacterial alanine-serine-cysteine exchanger (BasC) has been solved very recently, this template doesn’t fit our target homology models (see below)^20^. This lack of success in crystallizing human Asc-1 is of little surprise given the historic difficulties in solving the structures of membrane-bound proteins in general. To help circumvent this issue, we resorted here to computational techniques as a means for better understanding the potential conformational changes experienced by Asc-1 during the transport cycle. We first built homology models and docked d-serine. We then used molecular dynamics simulations to calculate possible transitions between the different conformations and to better understand the processes by which this transporter carries a substrate from the extracellular space to the cytoplasm across the cell membrane (Fig. 1). We then used the Asc-1 homology model to dock two known competitive inhibitors, (+)-amino(1-(3,5-dichlorophenyl)-3,5-dimethyl-1H-pyrazol-4-yl)acetic acid (ACPP) and LuAE00527^8,16^. Both compounds were fitted at the binding site showing interactions with TM1, TM6 and TM8 thus blocking the rocking movement of the substrate translocation. We also docked S-methyl-L-cysteine (SMLC) which is a competitive Asc-1 inhibitor that blocks the d-serine uptake but not its efflux^8,21^. We show that the mobility of Phe243 strongly influences that property. These results may support a new approach in drug design.

## Methods

All the preparation steps, calculations and analysis were performed in BIOVIA Discovery Studio 2018 and 2019 (BIOVIA Dassault Systèmes, Vélizy-Villacoublay, France).

### Homology models

The human amino acid sequence of Asc-1 (SLC7A10) used in this study was retrieved from UniProt Database (http://www.uniprot.org) under the code Q9NS82. At the time our studies were conducted, a multiple sequence alignment with NCBI BLAST on the protein data bank (PDB) identified bacterial transporters AdiC (PDB ID: 5J4I), GadC (PDB ID: 4DJI), MjApcT (PDB ID: 3GIA) and the recent release of the bacterial cationic amino acid transporter GkApcT (PDB ID: 5OQT) as the most homologous templates^22–25^.

The best alignment scores were obtained with both AdiC and GkApct templates with 18% sequence identity and 40% sequence similarity. In the case of AdiC, several crystal structures at high resolution were available as substrate-free in the outward state, but also co-crystallized with different substrates (PDB ID: 3LRB, 3NCY, 30B6, 3L1L, 5J4I, 5J4N)^22,26–29^. On the contrary, no crystal structure of GkApcT was solved in the apo outward-open state, and the substrate-bound GkApcT complex (PDB ID: 5OQT) was crystallized in the inward occluded state with the intracellular side noticeably more open^25^. Five other templates with better sequence identity scores, were very recently resolved but in the inward-open states. Among these new templates, two are bacterial including apo BasC (PDB ID: 6F2G) and BasC bound to α-aminoisobutyric acid (AIB) substrate (PDB ID: 6F2W). The others are human LAT1 linked to the heavy-chain including LAT1-CD98hc (PDB ID: 6JMQ) and LAT1-4F2hc (PDB IDs: 6IRS, 6IRT) and were solved by Cryo-EM^20,30,31^.

Both our three-dimensional (3D) structures of the Asc-1 outward-open model (substrate-free) and in complex with d-serine (outward-open occluded) were based on the sequence alignment of the crystal coordinates of the best resolved AdiC structures (PDB ID: monomer A of 5J4I and 3L1L, respectively). Our 3D structure of Asc-1 inward-open model was based on sequence alignment of the structure coordinates of both human LAT1-4F2hc (PDB ID: 6IRS chain B) and bacterial BasC bound to AIB with sequence identity of 47% and 26%, respectively. Therefore, human LAT1-4F2hc was chosen as the best template given its higher sequence identity.

During the sequence and structural alignment, special attention was paid to preserve the helical topology, and the gaps were manually positioned out of the secondary structure elements. Models were built with MODELER 9.0 as implemented in Discovery Studio. Missing loops were also generated and refined using an optimized MODELER protocol^32^.

### Docking substrates and inhibitors in the generated models

Substrates and inhibitors were docked into the Asc-1 models using GOLD as implemented in BIOVIA Discovery Studio 2018 and 2019 with flexible side chains located in the potential binding site, namely, Ile53 and Ser56 from TM1 and Phe243, Ser246, Ala244, from TM6^33^. When using the Arg-bound AdiC template (PDB ID: 3L1L), the arginine was removed and replaced by substrates. First, we kept only the poses that showed interactions with the conserved residues from Arg-bound AdiC and Ala-bound GkApcT complexes. Then, we used the fitness score to select the best poses and submitted them to energy minimization for refinement purposes.

### Molecular dynamics simulations

MD simulations were carried out on the Asc-1 model in the outward-open state complexed with d-serine. The Asc-1 complex was oriented according to the OPM database before being inserted into a lipid bilayer to mimic physiological conditions, using the CHARMM-GUI webserver (http://www.charmm-gui.org)^34,35^. We chose to use a heterogeneous bilayer by adding cholesterol to the POPC lipid in order to regulate the fluidity of the membrane.

The replacement strategy was chosen to generate the membrane around the protein. To fully solvate the protein, additional water molecules were placed on the top and bottom of the protein. In all systems, the salt (NaCl) concentration was set at 0.15 M, then the net and counter ions (sodium and chloride) were added to achieve charge neutrality. In the case of the outward-open Asc-1 model, each molecular system comprised approximately 32,600 atoms, including 77 lipid molecules and 5241 water molecules. The box size was about 65 × 65 × 85 Å^3^.

Several cycles of the steepest descent procedure and ABNR (Adapted Basis set Newton Raphson) were performed to optimize the structure. The system was heated progressively to 303 K, then equilibrated as a canonical ensemble (NVT) at 303 K. The standard six step CHARMM-GUI equilibration protocol for membrane proteins was conducted.

One to five independent 50–100 ns runs of unrestrained isothermal isobar ensemble (NPT) were performed for the production dynamics and were carried out at 303 K, using 1 atm with 2 fs time steps. All calculations were performed using the CHARMM36m force field^36,37^.

## Results and Discussion

### Goals and strategy overview

The main goal of our study was to generate hypotheses for the first steps of the transport mechanism of Asc-1 that could shed light onto amino acid uptake and potential modes for inhibition. Our approach was to first assimilate all relevant information known for Asc-1 and its close clustering members. This included the consideration of the general knowledge of transporter shuttle mechanisms, three-dimensional structures of related proteins, mutagenesis studies, and putative transition between conformational states. Once assembled, we carefully built homology models of the three-dimensional states of Asc-1 including the apo structure (“outward-open model”) and that of Asc-1 bound to d-serine (“bound occluded model”) and docked d-serine in these models. We then explored the very early steps of the substrate translocation. To better understand how this transporter carries a substrate from the extracellular space to the cytoplasm, we carried out further studies considering large structural rearrangements over a suitable time-scale. We finally docked a series of competitive inhibitors that provided new insight for the design of improved inhibitors.

### General knoweldge of Asc-1 transporter shuttle mechamisms

To shuttle amino acids, specific transporters undergo different states that alternately expose a substrate binding site to either side of the membrane. Crystallography and more recently Cryo-EM have lent support to this model by resolving three-dimensional structures in distinct conformations, including the occluded conformations where the amino acid is stacked within the binding site of the complex but also “outward-open” and “inward-open conformations” in which the substrate binding site is accessible to the extracellular and intracellular domains, respectively^28^.

Asc-1 is a sodium-independent, small, neutral amino acid exchanger whose substrate translocation is comparable to that previously described for ‘5 + 5 TM pseudo two-fold symmetry’ SLC7 members (only TM1 and TM6 with their respective TM1a, TM1b, TM6a, TM6b half-helices are shown in Fig. 1b). Transport of d-serine including the efflux of the substrate along with its uptake (indicated with red arrows) across the membrane involves roughly three states (Fig. 1b): outward-open (OO), occluded (O) and inward-open (IO) states with additional intermediate states such us outward-open occluded (OOO) and inward-open occluded (IOO) (not shown). The black hexagon represents the conserved gating aromatic amino acid in TM6. In the present study, we focused on the first steps of the Asc-1 transport mechanism, especially the transition between the outward-open (OO) and outward-open occluded (OOO) states, and the important gating role of an aromatic residue of TM6 in this process.

### Asc-1 transporter family

Heteromeric amino acid transporters (HATs) are composed of two subunits, including the light subunit (SLC7 solute light carrier 7 family) and the heavy subunit which is a disulfide-linked N-glycosylated type II membrane glycoprotein (SLC3 family)^30,38^. The light subunits of HATs called the L-type amino acid transporters (LAT) belong to the large amino acids, polyamines, and organic cations (APC) family of transporters. The light subunit is the catalytic component of these transporters whereas the heavy subunit seems to be important for trafficking to the plasma membrane^30,39,40^. Human Asc-1 is the light subunit (SLC7A10) and belongs to the LAT subfamily and is bridged through Cys154 to 4F2hc heavy subunit^30,41–43^.

### Sequence alignments and building two homology models

Our aim was to build two complete Asc-1 homology models of the states, “outward-open” and “outward-open occluded” conformations. Given the lack of X-ray crystal structures involving Asc-1 in OO and OOO states, homology models were derived from the well-resolved multi-states of the related AdiC transporter which served as structural templates. Despite the low sequence identity within the SLC7 family, these transporters generally consist of 12 transmembrane helices (TM) which adopt a conserved 5+5 inverted topology fold with a symmetry between (TM1-5) and (TM6-10) helices^44^. The two C-terminal helices, TM11 and TM12 wrap around the core of the protein. Both TM1 and TM6 are discontinuous and consist of two short alpha-helices (TM1a, TM1b; TM6a, TM6b) connected by a highly conserved unwound segment which is involved in the substrate binding site and therefore in the transport translocation mechanism described (Fig. 1).

Also of interest, the TM domain structures of the bacterial cationic amino acid transporter (APC) have been solved for GadC, MjApcT and GkApcT^23–25^. From this APC superfamily, the SLC7 subfamily is divided into two groups: the cationic amino acid transporters (CATs, SLC7A1−4 and SLC7A14) and the L-type amino acid transporters (LATs, SLC7A5−13 and SLC7A15)^45^. In addition, the *E.coli* arginine/agmatine antiporter AdiC are solved in multiple distinct conformations including the outward-open, outward-open occluded and inward-open states^26–29^.

**Figure 2:**
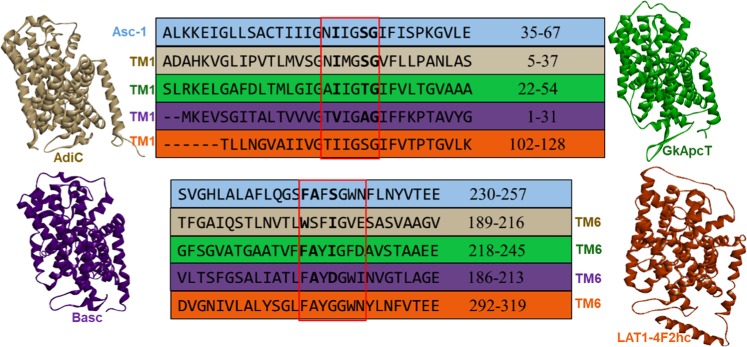
Partial sequence alignment of Asc-1 (blue) versus AdiC (PDB ID: 5J4I, chain A colored in beige), GkApcT (PDB ID: 5OQT, chain A colored in green), BasC (PDB ID: 6F2W colored in violet) and apo human LAT1–4F2hc (PDB ID: 6IRS, chain B colored in orange) showing conserved regions located in the TM1 and TM6 helices. The amino acid numbering scheme is based on the individual proteins. Unwound regions of TM1 and TM6 (framed in red) are very well conserved in the five sequences (more than 50% SI). The amino acids indicated in bold are involved in conserved interactions with the substrate within the binding site.

Based on the amino acid identity and similarity scores, a sequence alignment search reported, at the time our studies were conducted, the existence of two related proteins that were crystallized: AdiC (LAT, arginine agmatine exchanger, PDB ID: 5J4I) and GkApcT (CAT, a bacterial cationic amino acid transporter, PDB ID: 5OQT) with relatively low scores of approximately 20% for sequence identity (SI) and 40% for sequence similarity (SS) but with a high SI score (54%) in the conserved unwound regions (Fig. 2)^22–25^. Five recent structures (see details in the Methods section) with better sequence identity scores were published but these potential templates were solved in the inward-open state^20,30,31^. Sequence comparison of the human Asc-1 versus bacterial BasC bound to AIB substrate (PDB ID: 6F2W) and versus the human apo LAT1-4F2hc (PDB ID: 6IRS) show 26% and 47% of sequence identity, respectively. Partial sequence alignment of these two templates among the recent resolved structures are presented in Fig. 2.

In the present study, we focused on the outward-open and outward-open occluded states because they were crucial for understanding both the substrate uptake and the binding mode of the competitive inhibitors.

Although the alignment scores were comparable with both AdiC and GkApct templates, we decided to focus on the former since AdiC has been used as a model system for studying substrate translocation. Also, no crystal structure of GkApcT in the apo outward-open state was available and the substrate bound GkApcT complex (PDB ID: 5OQT) was crystallised in the inward occluded state with the intracellular side noticeably more open. In addition, six structures of AdiC are available including the wild-type (PDB IDs: 3LRB, 3NCY, 5J4I, 5J4N) and mutant (PDB IDs: 3L1L, 3OB6) conformations, in apo state (substrate-free) or complex in outward-open (OO) or outward-open occluded (OOO) states (Table 1)^22,26–29^.

**Table 1:** X-ray crystallographic structures of AdiC in different states are indicated along with respective PDB codes and resolutions.

**AdiC** PDB ID	State	Resolution (Å)	Reference
**5J4I**	apo (OO)	2.2	22
5J4N	Agm-bound (OO) complex	2.6	22
3LRB	apo (OO)	3.6	26
3NCY	apo (OO)	3.2	27
3OB6	Arg-bound (OO) complex N101A	3.0	28
**3L1L**	Arg-bound (OOO) Complex N22A; L123W	3.0	29

**Figure 3:**
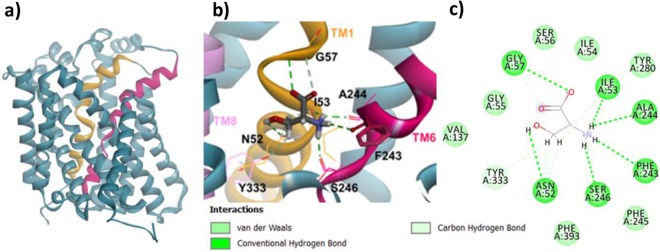
Homology model of Asc-1 in the outward-open conformation before and after docking d-serine. TM1 et TM6 are colored in yellow and raspberry pink, respectively. (**a**) Side view of the OO conformation. (**b**) Zoom on the top view of the OO conformation after docking d-serine. Numerous key interactions are observed between the backbone of both TM1 and TM6 and the amino acid moiety of the substrate. Specific vdw and H-bound contacts are also observed between TM1 and TM8 with d-serine side chain. (**c**) A 2D diagram of interactions between the Asc-1 OO model and d-serine.

**Figure 4:**
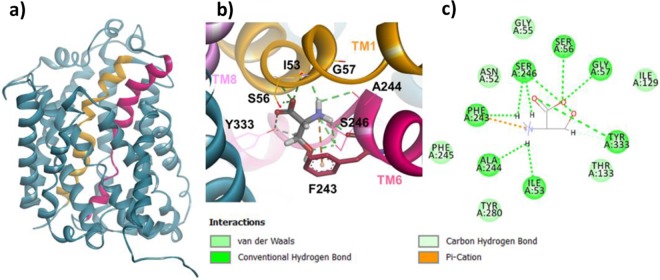
Homology model of Asc-1 in the outward-open occluded conformation before and after docking d-serine. TM1 et TM6 are colored as in Fig. 3. (**a**) Side view of the OOO conformation. (**b**) Zoom on the top view of the OOO conformation after docking d-serine. Similar bindings are observed as in Fig. 3 between the backbone of TM1, TM6, TM8 and the amino acid moiety of the substrate. Specific vdw and H-bound contacts are also observed between TM6, TM8 and d-serine side chain. **(c)** A 2D diagram of interactions between the Asc-1 OOO model and d-serine.

Given this critical information, the distinct templates from AdiC in the OO (PDB ID: 5J4I) and OOO (PDB ID: 3L1L) states were used to build Asc-1 homology models to reveal insights into potential transitions between the states. Two Asc-1 3D-models were built for the outward-open state and for the outward-open occluded state. These were based on the sequence alignment of the best resolved structures PDB ID: 5J4I and PDB ID: 3L1L, respectively (Figs. 3a, 4a)^22,29^.

As expected, both outward-open and outward-open occluded models (Figs. 3a, 4a) adopt a conserved pseudo-two-fold symmetry 5+5 (TM1-5 + TM6-10) with the two last helices, TM11 and TM12 wraping around the core of the protein. Both TM1 (yellow) and TM6 (raspberry pink) are discontinuous and consist of two short alpha-helices (TM1a, TM1b; TM6a, TM6b) connected by a highly conserved unwound segment which is involved in the binding site^28^.

### Dockings of d-serine in the OO and OOO 3D-models

To build the d-serine complexes with Asc-1 for the outward-open and outward-open occluded states, the GOLD program was used^33^. Fitness scores were evaluated to identify the best poses for d-serine in the putative binding site based on literature reports and template structures. For the complex involving d-serine bound to Asc-1 in the outward-open model, the structural template of the outward-open AdiC state was used (PDB ID: 5J4I). The docking of d-serine in the outward-open occluded Asc-1 model was performed based on the arginine-bound AdiC structural template (PDB ID: 3L1L), after removing the arginine substrate. The best docking poses were further refined by optimizing the interactions between d-serine and the protein binding pocket. It is interesting to note that the d-serine substrate bound in the outward-open model makes interactions to the backbone atoms of Asc-1 in unwound regions of TM1 and TM6. The amino group makes polar interactions to the carbonyl groups of Phe243, Ala244 and Ser246 from TM6 and Ile53 from TM1. On the other hand, the carboxyl makes polar contacts to the amido groups of Ser56 and Gly57 from TM1. Other specific interactions of d-serine side chain were observed including van der Waals (vdw) contacts with Asn52, Ile53 from TM1 and Tyr333 (TM8) and a hydrogen bond interaction with Asn52 (TM1) (Fig. 3b,c).

In the case of the Asc-1 complex in the outward-open occluded conformation, the interactions between the substrate and the backbone were identical to those in the OO model, with additional contacts between the carboxyl and Tyr333 hydroxyl (TM8) and between the amino group and Phe243 ring (Pi – cation). In contrast, the d-serine side chain makes different hydrogen bound and vdw contacts with Ser246 (TM6) keeping the one with Tyr333 (TM8) (Fig. 4b,c).

**Figure 5:**
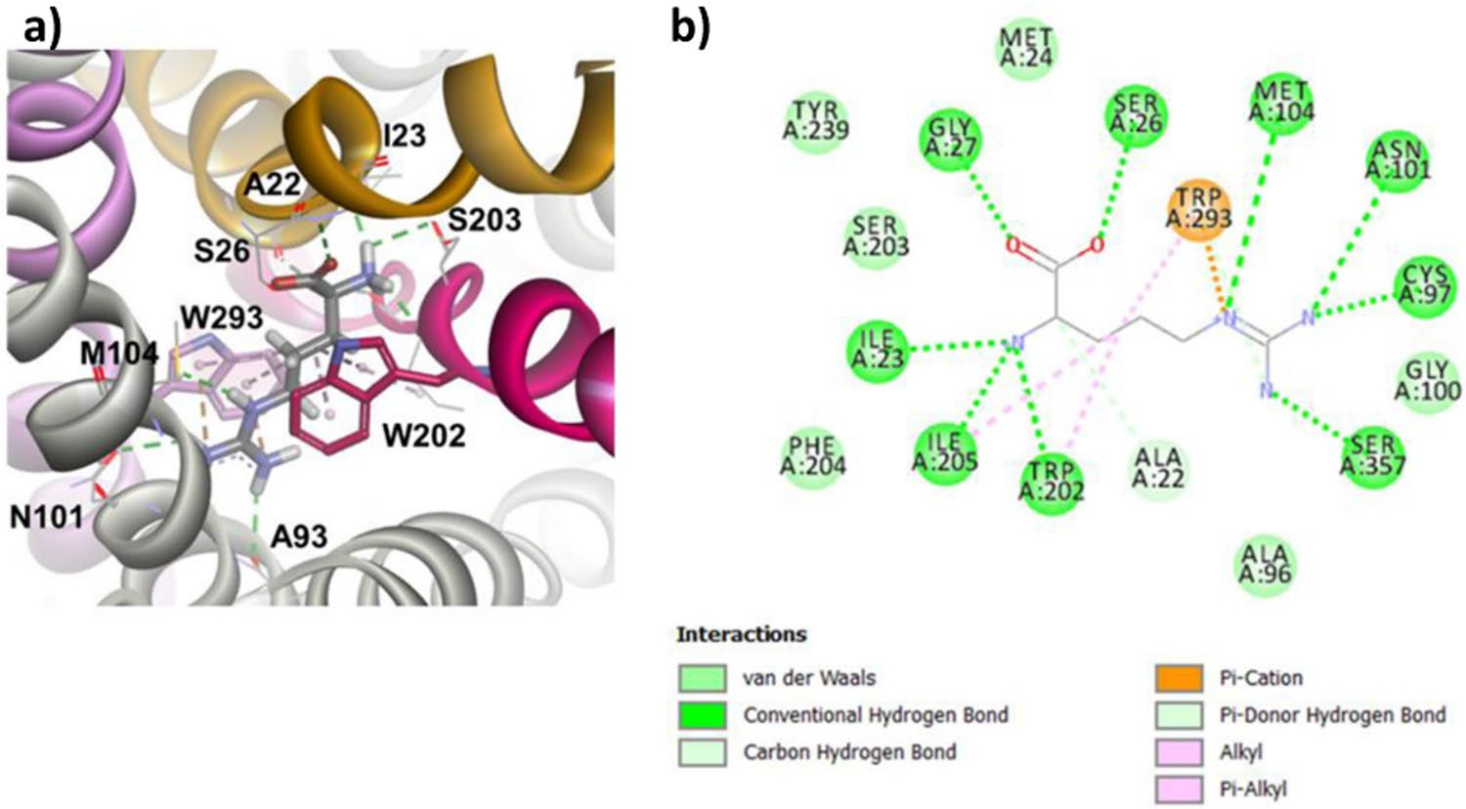
AdiC N22A and L123W (3L1L) structure in outward-open occluded (OOO) state bound to Arginine. (**a**) Zoom on the top view of the OOO conformation bound to the substrate. Key contacts are observed between the backbone atoms of the conserved residues of TM1 (yellow) and TM6 (raspberry pink) and the amino acid moiety of the substrate. The side chain of the Arginine substrate is sandwiched between Trp202 (TM6) and Trp293 (TM8) and the guanidinium makes several H-bonds with the backbone of TM3 residues. (**b**) 2D diagram of interactions between the AdiC OOO structure and Arginine.

The helix-breaking motifs in the binding site are well conserved in Asc-1 and AdiC (PDB ID: 5J4I) but also in GkApcT (PDB ID: 5OQT) (Fig. 2). This is consistent with Gao *et al*. 2010 who reported for l-Arg-bound outward-open occluded AdiC crystal complex (PDB ID: 3L1L) that the positively charged α-amino group of the arginine substrate donated three hydrogen-bonds to the carbonyl oxygen atoms of Trp202 and Ile205 in TM6 and Ile23 in TM1. Also, the α-carboxylate group of the ligand accepts two hydrogen bonds from the side chain of Ser26 and the amide nitrogen of Gly27 (Fig. 5)^29^. Both of these residues were located on the helix-breaking GSG conserved motif of TM1. Recently, the corresponding interactions in l-Ala-bound inward-open occluded GkApcT (PDB ID: 5OQT) were also reported (not shown)^25^. Within the binding site, numerous key interactions are observed between the l-alanine substrate and the GkApcT backbone atoms. The amino group makes polar interactions to the carbonyl groups of Phe231, Ala232 and Ile234 from TM6 and Ile40 from TM1, whereas the carboxyl group interacts with the amido groups of Thr43 and Gly44 from TM1. The recent bacterial structure BasC in the IO state, bound to AIB (PDB ID: 6F2W) also shows similar interactions in the binding site between the amino group of AIB and the carbonyl group of Ala200, Asp202 from TM6 and Val17 from TM1. In contrast, the carboxyl group of the substrate makes polar contacts with the amido groups of Ala20, Gly21 and an additional contact with the hydroxyl group of Y236. These key residues that are involved in this specific binding site are reported in bold for each respective sequence of the corresponding protein in Fig. 2.

When checking the position of Phe243 in both the Asc-1 OO and OOO models, we noted two different orientations of its side chain which is in agreement with the corresponding residue Trp202 in AdiC templates (Figs. 3b, 4b, 5)^28^. Indeed this key aromatic residue flips from an “out to in” position with respect to the binding site (Fig. 1b). The flipping of this residue will be discussed in the next paragraph.

### Simulating transitions

**Figure 6:**
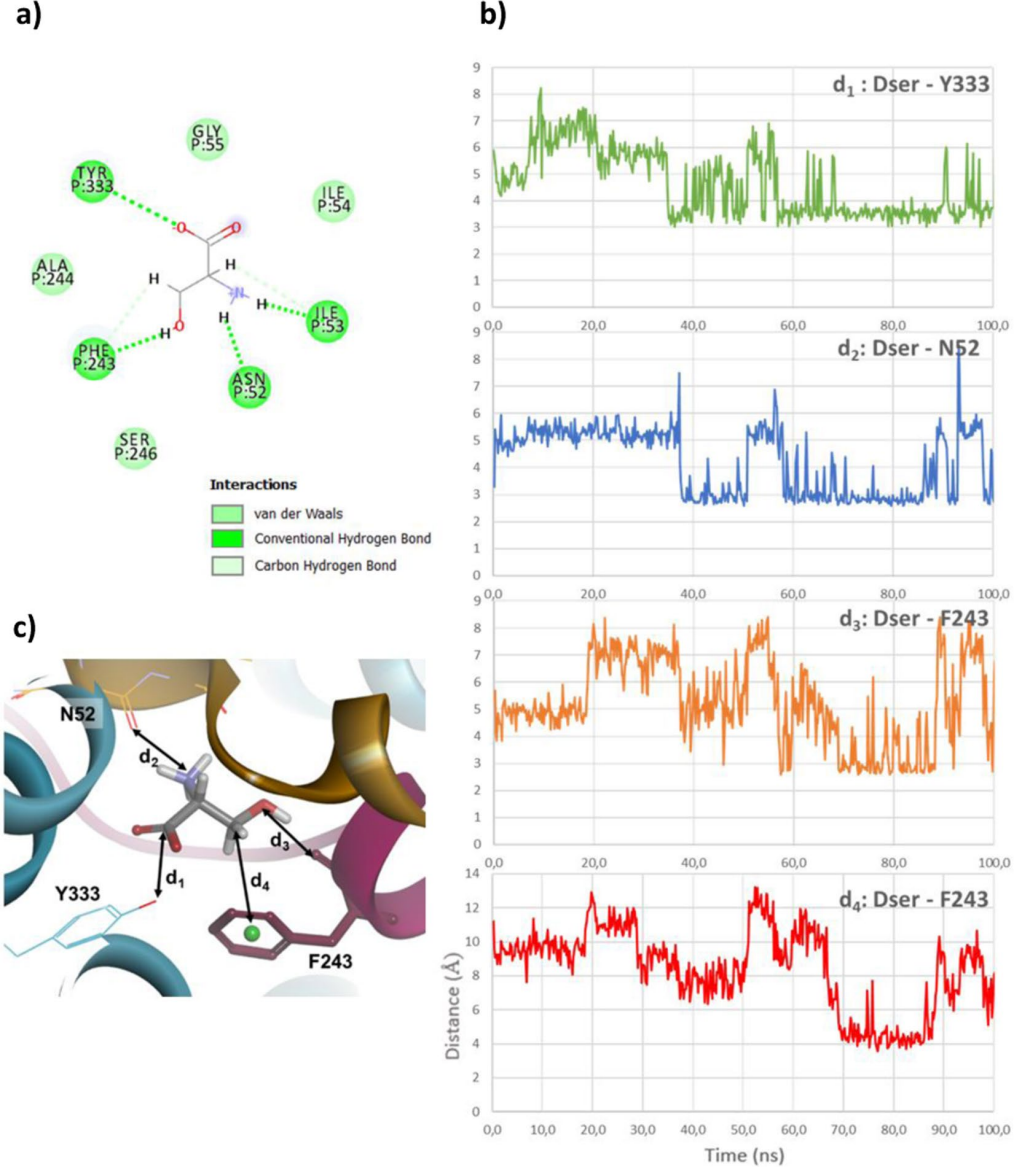
Asc-1 Interactions observed in the “transition” state. (**a**) 2D diagram of interactions between d-serine and Asc-1. (**b**) Monitoring of the distance (Å) between the carbon of d-serine carboxylate group and the oxygen of the Tyr333 hydroxyl group (d_1_, green), between the d-serine nitrogen and Asn52 main chain oxygen (d_2_, blue), between d-serine side chain oxygen and Phe243 main chain oxygen (d_3_, orange), between the carbon of d-serine side chain and centroid of Phe243 phenyl ring (d_4_, red). (**c**) Snapshot of MD simulation at 84 ns showing a “transition” state conformation with d1-d4 distances indicated.

After having built the homology models of the distinct states and complexes, we then focused our attention on simulating the early steps of the transition between Asc-1 outward-open conformation and Asc-1 outward-open occluded in complex with d-serine substrate. For this, we ran 100 ns molecular dynamic simulations (5 times) starting from the d-serine docked in Asc-1 outward-open model. From the 5 trajectories, 4 remained in the initial outward-open conformation and 1 exhibited a rearrangement at the binding site. We then explored the interactions between the protein and d-serine for the backbone and side chain atoms by monitoring relevant distances involving TM1, TM6 and TM8. We first monitored the carboxylate of d-serine and found that interaction to Gly57 (TM1) was lost in favor of Tyr333 (TM8) (d_1_). The amino group lost an interaction to Phe243 and Ala244 (TM6), kept the contact with Ile53 (distance not shown) and gained a hydrogen bond with Asn52 (d_2_). The side chain hydroxyl interacts with Phe243 (d_3_) instead of Asn52. The methylene protons of this side chain make hydrophobic contacts to the phenyl ring of this residue (d_4_) (Fig. 6). The analysis of these distances (d_1_–d_4_) shows they are all the shortest in the interval 69 ns-88 ns of MD, which seems to reveal an intermediate “transition” state.

**Figure 7:**
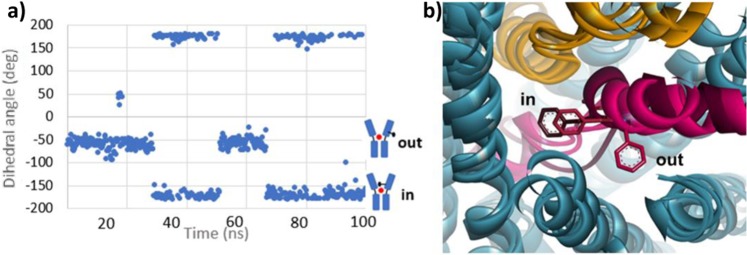
Movement of Phe243 aromatic ring during MD simulation. (**a**) Monitoring of the Phe243 dihedral angle N-Cα-Cβ-Cγ during the 100 ns MD simulation. A cartoon of the two “in” and “out” Asc-1 states describes the positions of Phe243. The “out” state corresponds to the outward-open (OO) Asc-1 state described in Fig. 1b while the “in” state corresponds to the “transition” state. (**b**) Superimposition of the three Asc-1 OO, OOO, “transition” states. Zoom on the different orientions of Phe243. TM1 and TM6 are colored in yellow and raspberry pink, respectively. TM6 including Phe243 is colored in darker pink in the “transition” state.

We observed a different distribution among the interactions in the binding site as compared to those observed in the OO initial model (Fig. 3b,c). Indeed d-serine changed its orientation based on the three functional group positions (Fig. 6a). When the early “transition” state was reached, the benzyl chain of Phe243 flipped around the unwound part of TM6. When we monitored its movement (dihedral angle N-Cα-Cβ-Cγ) during the 100 ns MD simulation, two distinct orientations were mostly observed “out” and “in”. The “out” conformation is oriented outside the binding site and has a mean dihedral angle of ~−60°. The “in” conformation is oriented towards the binding site and has a mean dihedral angle of ~−170° (Fig. 7a). The “out” orientation occurred twice (0–29 ns, 51–66 ns) as well as the “in” orientation (29–51 ns, 66–100 ns). Before 29 ns, the interaction network remained similar to the starting OO model. During 29–51 ns and 51–66 ns intervals, Phe243 flipped in then out but did not make favorable contacts with the substrate. Then it flipped in again (66–100 ns) and allowed the stabilizing interactions described above. This interval includes the time during which the “transition” state is observed. A similar study performed with l-alanine, l-serine and glycine substrates^4^ also shows the Phe243 flipping (see Supplementary Information, Fig. S1).

The analogous residue to Phe243 in AdiC is Trp202 (TM6) which has been shown to be essential for the transport activity of AdiC^22,28,29^. Comparison of AdiC in the outward-open state (PBD ID: 5J4I) and outward-open occluded Arg-bound AdiC (PDB ID: 3L1L) state also revealed major conformational changes, where the entire TM6a (half helix) flips around the unwound regions driving Trp202 to interact with the hydrophobic portion of the arginine substrate and blocking the exit route back to the extracellular space^28^. We observed a similar trend in our Asc-1 outward-open occluded model where the Phe243 functions as a gatekeeper (Fig. 4b). Interestingly, this particular residue, in our simulation (“transition” state) adopts a similar orientation (Fig. 7b) although the overall TM6a movement did not occur. Our study suggests that the flip of Phe243 and the reorientation of d-serine in the binding site, may both precede the movement of the TM6 helix.

### Potential binding modes of competitive inhibitors

We focused on the OO and OOO states of Asc-1 and their transition to shed light on the initial steps of substrate translocation. We then investigated the potential modes of inhibition for competitive inhibitors. These compounds bind to the OO conformation at the substrate binding site discussed above since they are competitive.

**Figure 8:**
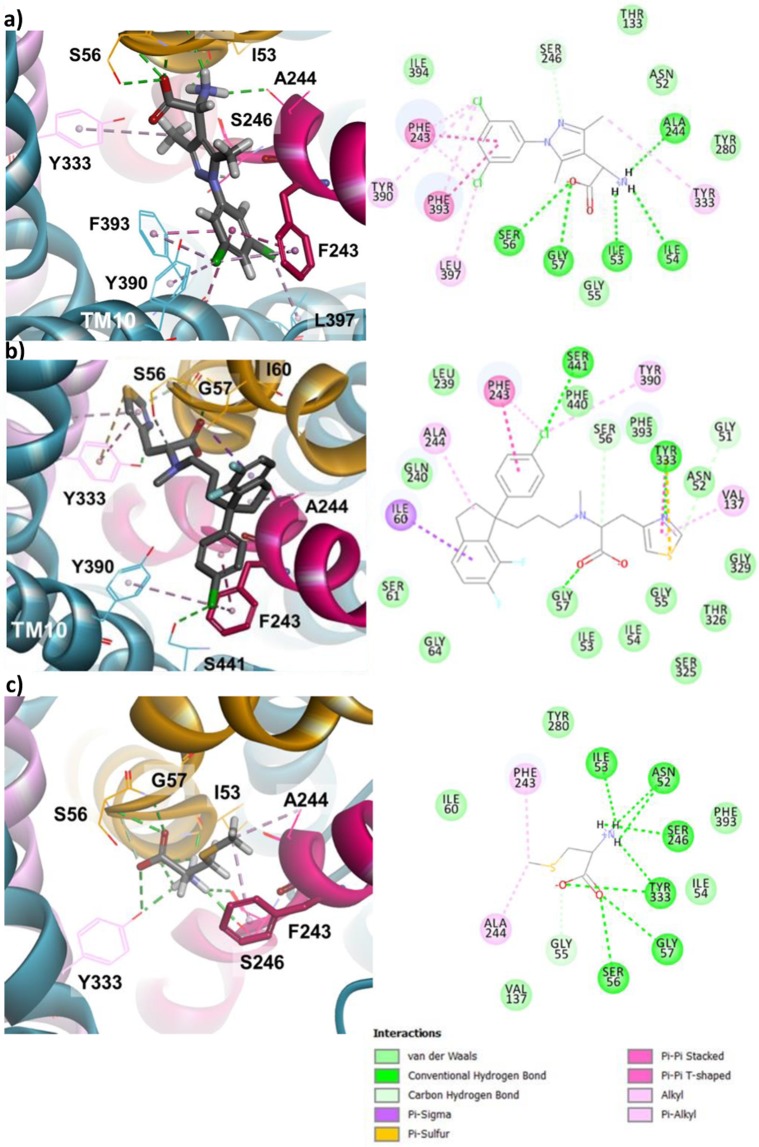
Docking 3 different inhibitors in the outward-open Asc-1 model. For each ligand, numerous vdw and H-bound contacts are observed with specific residues of TM1 (yellow), TM6 (raspberry pink),TM8 (pink) and TM10. 2D diagram of interactions between each ligand and Asc-1 are displayed in the right panels. (**a**) Docking (*D*)-ACPP in the OO Asc-1 model. (**b**) Docking LuAE00527 in the OO Asc-1 model. (**c**) Docking SMLC in the OO Asc-1 model.

We first docked at the substrate binding site, amino(1-(3,5-dichlorophenyl)-3,5-dimethyl-1H-pyrazol-4-yl)acetic acid (ACPP) which holds an amino acid moiety as d-serine (Fig. 8a)^8^. ACPP makes numerous contacts to the Asc-1 backbone atoms. The amino group predominantly interacts with the carbonyl groups of Ala244 from TM6 and of Ile53 and Ile54 from TM1, while the carboxyl group only interacts with the amido groups of Ser56 and Gly57 from TM1 (Fig. 8a). Meanwhile the dichlorophenyl scaffold is surounded by a cluster of aromatic residues (Phe243, Tyr390, Phe393) and in particular the phenyl ring is stacked between Phe243 the conserved aromatic residue of TM6 and Phe393 of TM10. The two chloro substituents also stabilize the system by interacting with Phe243, Tyr390, Phe393, L397 (Fig. 8a). The d-isomer fits better to the binding site than the l-isomer in our dockings. Whereas the amino acid motif of ACPP binds to Asc-1 in a similar mode as substrates (see Fig. 3a,b and 4a,b for d-serine), strong lipophilic interactions occur with its side chain (Pi-stacking and alkyl interactions, Fig. 8a). These later interactions stabilize the complex with ACPP in the outward-open conformation explaining the competitive inhibitory action of this compound. Indeed the tight interactions of the dichlorophenyl substituent prevent the mobility of Phe243 side chain. Consequently the movement of TM6 is blocked and translocation of substrates is not possible.

We also docked another potent competitive inhibitor (*R*)-2-({3-[(R)-1-(4-chloro-phenyl)-6,7-difluoro-indan-1-yl]-propyl}-methyl-amino)-3-thiazol-4-yl-propionic acid referred to as LuAE00527 which was recently described^16,21^. The binding site recognition of the amino acid moiety at TM1, TM6 and TM8 is provided by the carboxylic acid, the thiazole and indane rings. The carboxylic group forms a hydrogen bond with Gly57 (TM1), the thiaziole ring interacts with Asn52 (TM1), Val137 (TM3),Tyr333 (TM8) and the indane ring with Ile60 (TM1), Ala244 (TM6) (Fig. 8b). The chlorobenzene group is bound to Phe243 (TM6), Tyr390 (TM10), Ser441 (loop TM11-TM12). Similarly to ACPP this aromatic ring blocks the movement of Phe243 and thus the conformation switch that allows the substrate transport.

A related work described the docking of BMS-466442, an Asc-1 inhibitor, in a similar homology model^19^. However, this model was based on the same AdiC template but combined with a hSERT template in order to accommodate larger molecules. As seen with ACPP and LuAE00527, the ester moiety is bound to Ser56 (TM1) and Tyr333 (TM8) while the indole scaffold makes an aromatic interaction with Phe243 (TM6)^19^. Other interactions with residues from TM3, TM6 and TM10 further stabilize BMS-466442 in Asc-1 outward-open state. As seen for ACPP and LuAE00527 strong hydrophobic interactions block the mobility of Phe243 and consequently inhibit the transport process.

Another competitive Asc-1 inhibitor is S-Methyl-L-Cys (SMLC). Nevertheless ACPP, LuAE00527 and BMS-466442 block uptake and efflux of d-serine, while SMLC inhibits the uptake, but allows the efflux^8,16,18,19,21,46^. We performed the docking of SMLC at the binding site of our Asc-1 outward-open model. We observed that it binds similar to the substrates for the amino acid moiety and that the S-methyl side chain makes a hydrophobic contact with Phe243 and Ala244 of TM6 (Fig. 8c). The latter interaction is not as strong as seen with the previous inhibitors, thus, although it may interfere with the movement of TM6, it does not block it as ACPP and LuAE00527 do. We noticed that the side chain of Phe243 flipped from its initial conformation in the OO state (Fig. 8c). The flexible side chains in the GOLD docking allowed for the movement of that side chain. This situation where Phe243 is mobile, may explain the particular inhibitory property of SMLC compared to ACPP and LuAE00527. Another difference between SMLC and the other Asc-1 inhibitors, is that it is most likely translocated whereas ACPP and LuAE00527 are not. A trans-stimulation may then explain the increase of d-serine efflux upon SMLC extracellular application^8^. Asc-1 as many amino acid exchangers (antiporters) mediates the translocation of amino acids across the membrane in opposite directions with asymmetric features. The empty transporter return from the trans side to the cis side of the membrane limits the rate of efflux transport. However, the trans-stimulation may allow a faster return to the cis side when a substrate is bound to this same side. In other words, the presence of an extracellular substrate stimulates the efflux of another intracellular substrate. Therefore, it is expected that Asc-1 mediated efflux and uptake transport modes would not operate at the same velocity and with the same affinity for its substrates. Indeed, Bartoccioni *et al*. have shown recently that a close bacterial homolog of Asc-1 displays distinct apparent affinities for l-serine as a substrate by two orders of magnitude for the external and cytoplasmic sides^47^. Interestingly, the acceleration process due to the binding of an extracellular substrate has been used to discriminate between non transportable-inhibitors (“blockers”) and transportable-inhibitors (substrates) of LAT1^48–50^. Indeed, Geier *et al*. used the trans-stimulation assay to provide evidence that some novel inhibitors were actually substrates of LAT1^48^. Similarly, inhibitors that allow efflux of Asc-1 are most probably substrates of this transporter and accelerate substrate release from cells by trans-stimulation. Accordingly, SMLC allows efflux of Asc-1 substrates including d-serine as reported by Sakimura *et al*. due to trans-stimulation and is translocated as suggested by these authors^8^. Altogether, SMLC would show cis inhibition thus inhibiting substrates uptake while trans-stimulating the efflux. Likewise, this trans-stimulation may well explain why d-isoleucine which blocks uptake of d-serine by Asc-1, accelerates the release of d-serine from neural tissues^13^.

Several additional arguments support the substrate behavior of SMLC. First, neutral amino acids such as l-leucine, l-valine, AIB and l-methionine are substrates of Asc-1^4^. l-methionine is the homo-analogue of SMLC with the insertion of an additional -CH_2_- unit into the side chain. We can thus anticipate that SMLC, which is the shorter analogue of l-methionine, will be also transported by Asc-1. Moreover, the transport of SMLC is supported by our molecular modeling simulations. Asc-1 substrates that travel from extracellular space to the cytosol bind successively to the OO, OOO and IO conformations. This is illustrated with d-serine bound to the Asc-1 homology models of the OO (Fig. 3), OOO (Fig. 4) and IO (Fig. S3b,c) conformations. The homology model of the Asc-1 IO conformation and the related dockings were performed as described for the OO and OOO conformations using the LAT1–4F2hc template (PDB ID: 6IRS). Similarly SMLC was docked in the OO (Fig. 8c), OOO (Fig. S2), IO (Fig. S3d,e) conformations. In all three models, SMLC interacts with the same binding site residues as d-serine for the amino acid moiety. In addition, the side chain of SMLC makes hydrophobic contacts with the flipped Phe243 in all conformations. In particular, it fits nicely in the OOO model (Fig. S2) whereas the other inhibitors could not be accommodated in this more restricted site. Altogether, these compelling arguments support the substrate nature of SMLC which inhibits substrate (such as d-serine) uptake but allows their efflux. Such features of SMLC and transportable inhibitors also imply that their trans-stimulating efficiency in accelerating substrates efflux from cells is expected to be more pronounced at lower substrates concentrations, indicating they compete with the substrate for uptake but not for release^13^. A complete understanding of the Asc-1 inhibitory mechanism of SMLC may assist the design of new inhibitors with similar transport properties and potential therapeutic benefits.

In the present work, homology modeling, substrate docking and molecular dynamics simulations were performed. Our results provide insights into the initial steps of the substrate transport mechanism. We suggest that substrate translocation can be described by conformational transitions from substrate binding on the extracellular membrane (outward-open conformation), to a “transition” state before an outward-open occluded conformation and release on the intracellular side (inward-open conformation). We focused on the transition between the outward-open and outward-open occluded states as a means of exploring the first steps of the Asc-1 transport mechanism. These steps are critical for the design of therapeutic Asc-1 inhibitors that could prevent uptake of the substrates without blocking efflux.

When a substrate binds to Asc-1, the conserved gating aromatic residue Phe243 (TM6) closes on the top of the binding site thus blocking the substrate from the exit route back to the extracellular region, and initiating conformational changes of the protein. This first step of the transport mechanism is the one that is blocked by competitive inhibitors. We have demonstrated how ACPP, LuAE00527 and SMLC bind to the outward-open state of Asc-1 and the subtile difference for SMLC. Our results may open the field of new inhibitor discovery with fine-tuned therapeutic properties.

## Supplementary information

Supplementary Information.
