# Supplementary material for: Asc-1 Transporter (SLC7A10): Homology Models And Molecular Dynamics Insights Into The First Steps Of The Transport Mechanism
Source: Sci Rep. 2020 Feb 28;10:3731. doi: 10.1038/s41598-020-60617-y (PMC7048771; doi:10.1038/s41598-020-60617-y)
Supplement: Supplementary file 1 — Supplementary Information. [file 41598_2020_60617_MOESM1_ESM.pdf]

Supplementary Information

Asc-1 Transporter (SLC7A10): Homology Models And Molecular Dynamics Insights Into The First Steps Of The Transport Mechanism

Afaf Mikou[a*], Alexandre Cabayé[a,b], Anne Goupil[b], Hugues-Olivier Bertrand[b], Jean-Pierre Mothet[c] & Francine C. Acher[a*]

[a] Laboratoire de Chimie et Biochimie Pharmacologiques et Toxicologiques, UMR 8601 CNRS, Université de Paris, 45 rue des Saints-Pères, 75270 Paris Cedex 06, France
[b] BIOVIA, Dassault Systèmes, 10 rue Marcel Dassault, CS 40501, 78946 Vélizy-Villacoublay Cedex, France
[c] LuMIn FRE2036, Université Paris-Saclay, CNRS, ENS Paris-Saclay, CentraleSupélec, 91190, Gif-sur-Yvette, France

* Correspondence and requests for materials should be addressed to A.M. (email: afaf.mikou@parisdescartes.fr) or F.A. (email: francine.acher@parisdescartes.fr)

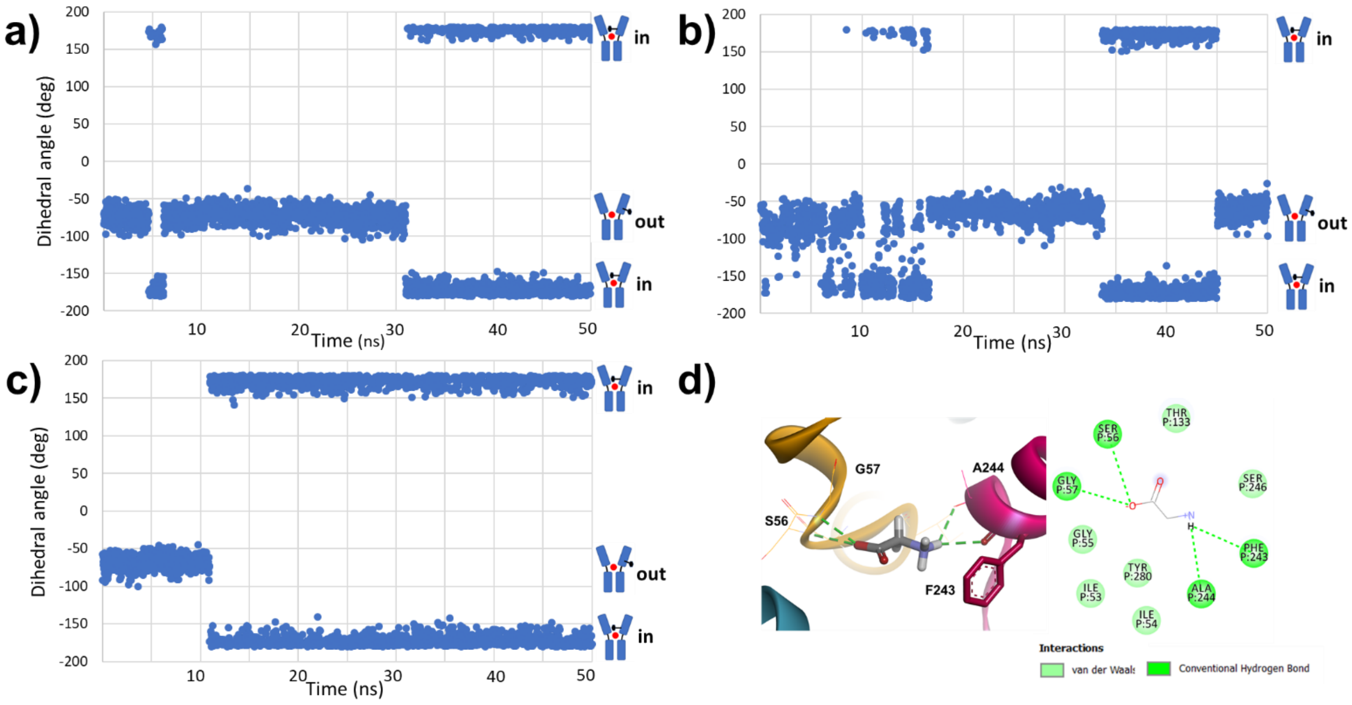

Figure S1. Monitoring the movement of Phe243 aromatic ring during molecular dynamic (MD) simulations of the complex Asc-1 in the OO conformation docked with three substrates: **a)** in the case of L-alanine, **b)** in the case of L-serine, **d)** in the case of glycine. The monitoring was based on the Phe243 dihedral angle N-Cα-Cβ-Cγ during the 50 ns MD simulations which were run several times for each substrate. The flip of Phe243 was observed once for L-Ala, once for L-Ser out of 3 MDs, twice for Gly out of 5 MDs. A cartoon of the two "in" and "out" Asc-1 states describes the positions of Phe243 as in Figure 7a. **d)** A snapshot of MD simulation showing the flip of Phe243 and the interactions between the glycine substrate and the Asc-1 TM1 and TM6. A 2D diagram of these interactions is displayed in the right panel.

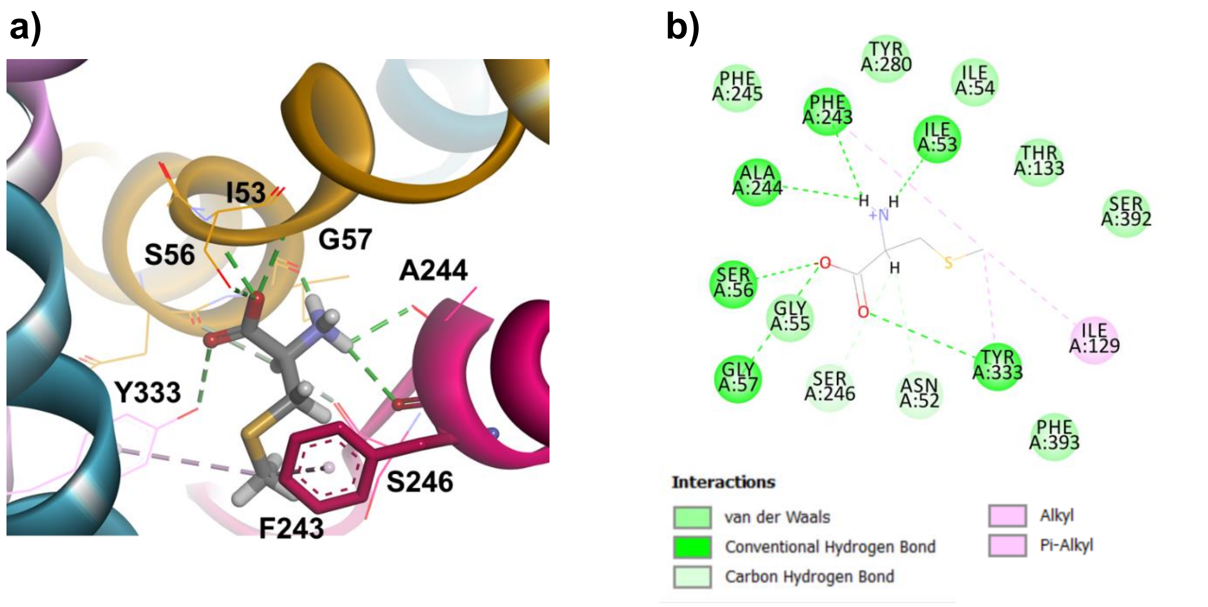

Figure S2. Homology model of Asc-1 in the outward-open occluded conformation docked with SMLC. TM1, TM6 and TM8 are colored in yellow, raspberry pink and light pink respectively. **a)**. Zoom on the top view of the OOO conformation after SLMC docking. Multiple key interactions are observed between the residues of both TM1 and TM6 and the amino acid moiety of SMLC. Pi-alkyl interactions are observed between TM6 (F243) and TM8 (Y333) and SMLC side chain. **b)** A 2D diagram showing the interactions between the Asc-1 OOO model and SMLC.

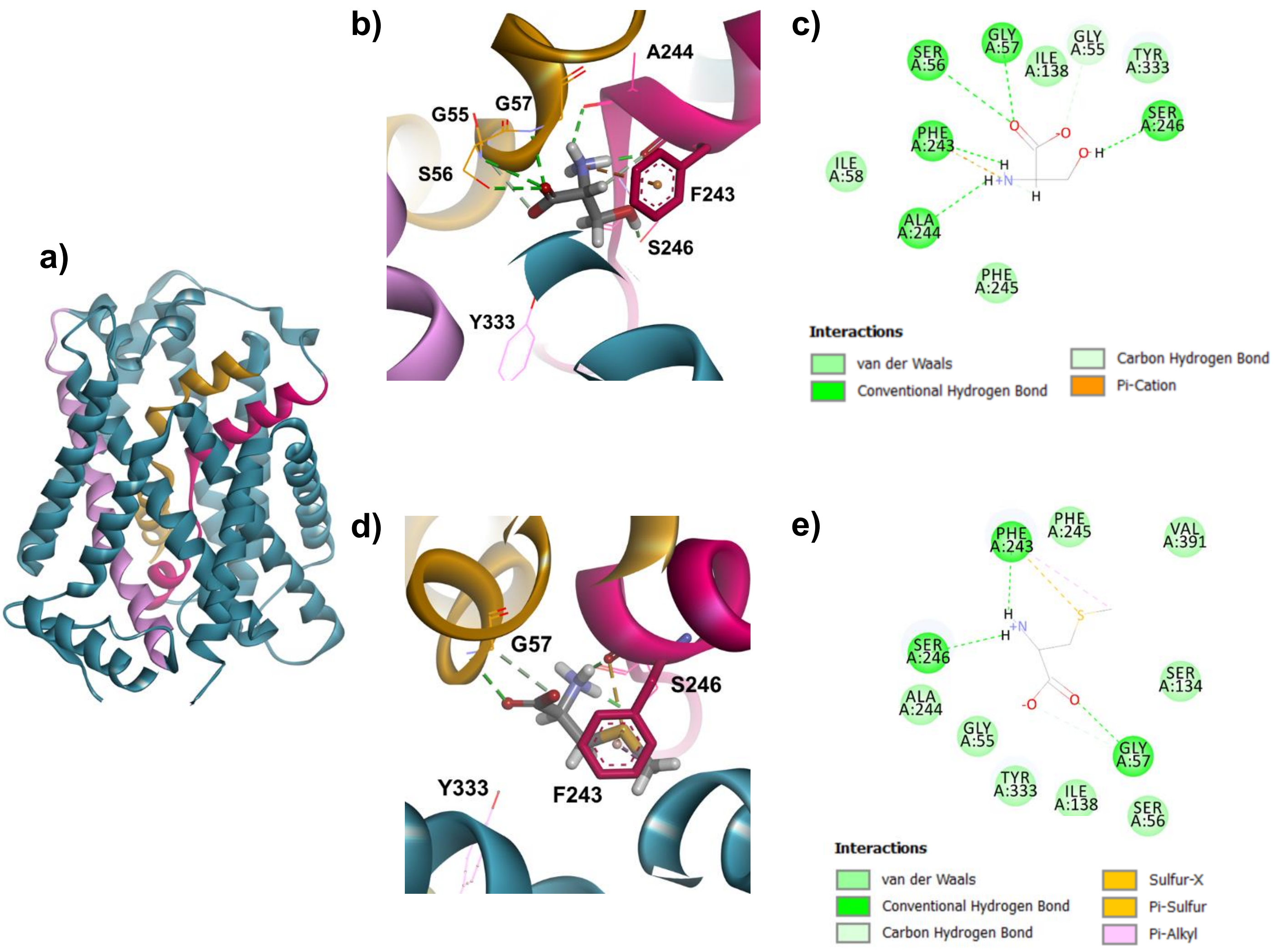

Figure S3. Homology model of Asc-1 in the inward-open (IO) conformation before and after docking D-serine and SMLC. TM1, TM6 and TM8 are colored in yellow, raspberry pink and light pink, respectively. **a)** Side view of the IO conformation before D-serine docking. **b)** Zoom on the top view of the IO conformation after D-serine docking. Conserved key interactions are observed between residues of both TM1 and TM6 and the amino acid moiety of the substrate. **c)** A 2D diagram of interactions between the Asc-1 IO model and D-serine. **d)** Zoom on the top view of the IO conformation after docking SMLC. Key interactions are observed between residues of both TM1 and TM6 and the amino acid moiety of SMLC. A specific Pi-alkyl interaction is also observed between TM6 and SMLC side chain. **e)** A 2D diagram of interactions between the Asc-1 IO model and SMLC. Similar conserved key interactions are observed in the recent bacterial BasC structure bound to AIB substrate (PDB ID: 6F2W). Among these conserved interactions, the carboxyl group of AIB interacts with TM1, making polar contacts to the amido groups of Ala20 and Gly21. In contrast, the amino group of AIB interacts with the carbonyl group of Val17 (TM1) and Ala200 and Asp202 (TM6). The TM1 and TM6 residue numbering (including the conserved residues at the binding site) is based on the individual proteins (Fig.2).
